# Changes in soil oxidase activity induced by microbial life history strategies mediate the soil heterotrophic respiration response to drought and nitrogen enrichment

**DOI:** 10.3389/fmicb.2024.1375300

**Published:** 2024-03-15

**Authors:** Weirong Zhuang, Yong Li, Xiaoming Kang, Liang Yan, Xiaodong Zhang, Zhongqing Yan, Kerou Zhang, Ao Yang, Yuechuan Niu, Xiaoshun Yu, Huan Wang, Miaomiao An, Rongxiao Che

**Affiliations:** ^1^Yunnan Key Laboratory of Soil Erosion Prevention and Green Development, Institute of International Rivers and Ecosecurity, Yunnan University, Kunming, China; ^2^Ministry of Education Key Laboratory for Ecosecurity of Southwest China, Yunnan University, Kunming, China; ^3^Beijing Key Laboratory of Wetland Services and Restoration, Wetland Research Center, Institute of Ecological Conservation and Restoration, Chinese Academy of Forestry, Beijing, China; ^4^Sichuan Zoige Wetland Ecosystem Research Station, Tibetan Autonomous Prefecture of Aba, Beijing, Sichuan, China; ^5^College of Life Sciences, University of Chinese Academy of Sciences, Beijing, China

**Keywords:** Qinghai-Tibet plateau, drought, N deposition, microbial community composition, microbial life history strategy, extracellular enzyme activity, heterotrophic respiration

## Abstract

Drought and nitrogen deposition are two major climate challenges, which can change the soil microbial community composition and ecological strategy and affect soil heterotrophic respiration (Rh). However, the combined effects of microbial community composition, microbial life strategies, and extracellular enzymes on the dynamics of Rh under drought and nitrogen deposition conditions remain unclear. Here, we experimented with an alpine swamp meadow to simulate drought (50% reduction in precipitation) and multilevel addition of nitrogen to determine the interactive effects of microbial community composition, microbial life strategy, and extracellular enzymes on Rh. The results showed that drought significantly reduced the seasonal mean Rh by 40.07%, and increased the Rh to soil respiration ratio by 22.04%. Drought significantly altered microbial community composition. The ratio of K- to r-selected bacteria (B_K:r_) and fungi (F_K:r_) increased by 20 and 91.43%, respectively. Drought increased hydrolase activities but decreased oxidase activities. However, adding N had no significant effect on microbial community composition, B_K:r_, F_K:r_, extracellular enzymes, or Rh. A structural equation model showed that the effects of drought and adding nitrogen via microbial community composition, microbial life strategy, and extracellular enzymes explained 84% of the variation in Rh. Oxidase activities decreased with B_K:r_, but increased with F_K:r_. Our findings show that drought decreased Rh primarily by inhibiting oxidase activities, which is induced by bacterial shifts from the r-strategy to the K-strategy. Our results highlight that the indirect regulation of drought on the carbon cycle through the dynamic of bacterial and fungal life history strategy should be considered for a better understanding of how terrestrial ecosystems respond to future climate change.

## 1 Introduction

Soil respiration (Rs) is one of the largest carbon (C) effluxes between terrestrial ecosystems and the atmosphere and plays an important role in regulating atmospheric CO_2_ concentration (Davidson et al., 2002; Kuzyakov, 2006; Baldocchi et al., 2018). Soil heterotrophic respiration (Rh) is mainly derived from the decomposition of litter and soil organic matter (Kuzyakov, 2006). However, the underlying mechanisms of the Rh response to climate change are uncertain (Ye et al., 2019). Soil microorganisms, the main decomposers of terrestrial ecosystems, are critical in terrestrial C cycling (Fierer, 2017; Banerjee et al., 2018). Environmental changes could alter C cycling via microorganisms (Davidson and Janssens, 2006; Romero-Olivares et al., 2017). Thus, disentangling the role of extrinsic factors and microbial mechanisms in driving Rh is imperative for predicting C cycling under future global change scenarios (Hashimoto et al., 2015; Hursh et al., 2017).

Climate change-induced extremes in the precipitation pattern are becoming more severe and frequent (Huang et al., 2017; Zhao and Dai, 2022), disrupting biogeochemical cycling in terrestrial ecosystems (Sippel et al., 2018; Xu et al., 2019; de Vries et al., 2020). Meta-analysis reported that moderate and extreme decreases in precipitation have significant negative effects on Rh in grasslands (Du et al., 2020). Previous studies have shown that drought changes the soil microbial community structure (Evans et al., 2014; Bastida et al., 2017; de Vries et al., 2018; Ochoa-Hueso et al., 2018). Many studies have shown that the soil microbial community shifts significantly with higher or lower aridity (Neilson et al., 2017; Tu et al., 2017; Yao M. J. et al., 2017; Song et al., 2019; Xu et al., 2020). Inputs of reactive nitrogen (N) from human activities, including combustion-related NO*_x_*, and industrial and agricultural N fixation, which are predicted to be 600 Tg N yr.^−1^ by 2,100, strongly affect ecosystem C cycling (Fowler et al., 2015). A global meta-analysis revealed that adding N reduces Rh and increasing the rate of adding N enhances reduction (Chen and Chen, 2023). N inputs eutrophy and acidify soil leading to altered microbial community structure and reduced Rh (Erisman et al., 2013). Moreover, adding N significantly shifts the microbial community structure, particularly in N-limited ecosystems (Sun et al., 2019; Zhou et al., 2022). However, N deposition simulation generally applied rates that much higher than the critical threshold of 10 kg N ha^−1^ yr.^−1^ (Dentener et al., 2006; LeBauer and Treseder, 2008; Xia and Wan, 2008; Bobbink et al., 2010; Janssens et al., 2010; Peng et al., 2017). Therefore, the interactive effect of drought and multilevel addition of nitrogen on the Rh should be fully investigated.

The life history strategies of soil microbes determine their metabolic potential and responses to environmental changes, thus driving the change in Rh (Piton et al., 2023). The r-selected species (copiotrophic) have a fast growth rate and a rapid response to available C and nutrient inputs and typically flourish in environments enriched in labile C. In contrast, K-selected species (oligotrophic) are slow-growing and more efficient species with recalcitrant C and lower availability (Fierer et al., 2007; Trivedi et al., 2013). A subtropical forest experiment showed that reducing throughfall increases the relative abundance of r-strategy bacteria, but decreases K-strategy bacteria (Yang et al., 2021b). A previous study revealed the dominant microbial growth strategy shifted from a K-strategy to an r-strategy in degraded grasslands after adding N (Zeng et al., 2021). The r-strategy-dominated soils generally have higher microbial respiration than K-strategy-dominated soils (Malik et al., 2016; Tosi et al., 2016). The rRNA operon (rrn) copy number correlates with the bacterial reproduction rate and the response rate of resource availability (Roller et al., 2016; Wu et al., 2017); thus, reflecting the ecological strategy of bacteria, as a higher rrn copy number is associated with faster growing copiotrophic or r-selected bacteria (Roller et al., 2016; Samad et al., 2017). Drought increases the mean rrn copy number, indicating a higher proportion of r-selection and average potential growth rate (Veach and Zeglin, 2020). A previous study showed that adding N promotes the abundance of bacteria with a higher rrn copy number, thus providing evidence that increased N input favors copiotrophic taxa (Liu et al., 2020; Ma et al., 2022). N deposition may enhance water limits by promoting plant growth, which in turn reduces the availability of soil microbial substrates and limits microbial growth and population size (Chen et al., 2021). In addition, drought in grasslands may further restrict nutrients and thus affect microbial communities (Yang et al., 2021a). However, to what extent the microbial life strategy contributes to the shift of Rh in response to the interaction between drought and adding N remains unclear.

Soil extracellular enzyme activities are central to Rh as they control the decomposition and mineralization of soil organic matter (Schimel and Bennett, 2004; Bengtson and Bengtsson, 2007). A previous alpine meadow study demonstrated that drought increases the activities of some hydrolases in some years, but non-significantly decreases the activities of oxidases (Yan et al., 2020). In another study, drought does not affect the activities of hydrolases but increases that of oxidases (Yan et al., 2021). Adding nitrogen also affects soil extracellular enzyme activities. A previous study on a steppe showed that depositing N decreases peroxidase activity by affecting environmental factors (Liu et al., 2018). In a 5-year field experiment on a meadow steppe in northern China, adding N increased the activities of α-glucosidase, and β-glucosidase (Ma et al., 2020). The lignocellulose index (LCI) can be calculated based on hydrolytic and oxidative activities and represents soil substrate C quality. The higher the LCI value, the more vulnerable substrate C is to being decomposed (Moorhead et al., 2013). A recent study reported that Rh and the LCI are positively correlated (Jiang et al., 2023). The definite pattern of soil extracellular enzyme activities response to drought and N deposition should be identified to predict Rh more accurately under global change.

The Zoige alpine wetland is located on the eastern edge of the Qinghai-Tibet Plateau and is the largest plateau peat swamp wetland in the world (Wu et al., 2020). Due to its altitude, it is highly sensitive to climate change (Yu et al., 2010; Chen et al., 2014; Zeng et al., 2017). The Zoige plateau plays an important role in the global C cycle (Wang et al., 2012). As such, this region could potentially have a significant impact on regional climate change (Kang et al., 2014). Here, we examined the effects of drought- and N-induced changes in microbial community composition, microbial life strategies, and extracellular enzyme activities on Rh across drought and multiple levels of added N in the Zoige alpine swamp meadow. We hypothesized that (1) the soil microbial community would change to copiotrophic taxon-dominated under the drought and added N conditions; (2) drought and adding N will decrease soil extracellular enzyme activities; (3) drought and adding N will decrease Rh. This study aimed to explore the underlying mechanisms of the responses of Rh to drought and adding N from the perspectives of microbial life strategy and extracellular enzymes.

## 2 Materials and methods

### 2.1 Site description and experimental design

This study was performed in a typical swamp meadow ecosystem at the Drought and Nitrogen Deposition Interaction Experiment Platform in Xiangdong village (33°37′16″N, 102°52′21″E, 3,400 m above sea level), Zoige county, Sichuan Province (northeastern Qinghai-Tibetan Plateau). The mean temperature in the region ranges from −0.7 to 1.1°C, with the coldest temperature in January at −10.5°C and the hottest in July at 11°C, respectively. The mean annual precipitation is 650–750 mm, which mainly occurs from June to September. The soil type is peat and swamp soil. The dominant plant species are *Poa poophagorum*, *Elymus nutans*, *Carex atrofusca*, and *Potentilla anserina*. Soil samples from the study site were collected before the experiment and analyzed. The soil properties of the top 0–20 cm layer were: pH 6.04, total organic C 66.8 mg g^−1^, total N 5.09 mg g^−1^, and total P 0.90 mg g^−1^.

In June 2019, a 90 × 90 m plot was enclosed with a 1.6-m-high fence, which prevented herbivores from entering. An interactive experiment between drought and N enrichment was established using a randomized complete block design with two levels of drought (CK and drought) and six levels of N enrichment (0, 2, 4, 8, 16, and 32 g N m^−2^ yr.^−1^). Four replicate blocks were established and each block had 12 treatments randomly assigned to 4 × 4 m plots. Each plot was 2 m away from neighboring plots. We fixed rainout shelters on the plots to simulate drought in September 2019 and each rainout shelter had a roof made of curved bands of transparent acrylic that intercepted 50% of the rainfall while having a minimal impact on other environmental factors (Zhang et al., 2019a). Iron sheets were installed around the plots to a depth of 40 cm belowground to prevent lateral movement of water between the plots (Zhang et al., 2019b). Six levels of N enrichment consisted of the range from the plant N limitation to saturation according to a previous study conducted near the experimental site (Song et al., 2017). At the end of the 2020 growing season, the effect of the drought treatment on soil water content was tested and was significant, so we initiated the N addition treatment in May 2021. Coated slow-release urea was spread onto the soil surface of the plots by hand at the beginning of the growing season each year.

### 2.2 Soil respiration measurements

Before the experimental treatments were initiated, soil respiration collars (PVC pipe, 20 cm inner diameter) were installed in the ground in each plot. Two soil respiration collars were set up per plot; one shallow (5 cm) for measuring Rs, and one deep (40 cm) for measuring Rh. The trench method was successfully used in previous studies (Hanson et al., 2000; Keeler et al., 2009; Sayer and Tanner, 2010).

Soil respiration (Rs) and heterotrophic respiration (Rh) were measured with a portable soil carbon flux automatic measurement system (PS-9000, LICA, Beijing, China) once every 2 weeks during the growing season. The above-ground parts of newly growing plants were cut off at the surface in both collars in advance. Soil temperature (ST) and soil water content (SWC) were simultaneously measured with a soil temperature and humidity probe on a portable soil carbon flux automatic measurement system in the 10 cm soil layer during the carbon flux measurement.

### 2.3 Soil property determination

At the end of August 2022, 48 soil samples were collected by drilling 0–20 cm soil layers in 48 plots, removing the stones, roots, and other impurities, and dividing them into two subsamples. Subsample 1 (10 g) was wrapped in foil, quickly placed in liquid nitrogen, transported back to the laboratory, and stored at −80°C for soil DNA extraction. Subsample 2 (200 g) was transported back to the laboratory and stored at −4°C to determine the soil physicochemical properties.

Soil-dissolved carbon (DOC) was extracted by adding 50 mL of 0.5 M potassium sulfate to subsamples of 12.5 g homogenized soil and agitating the sample on an orbital shaker at 120 rpm for 1 h. The filtrate was analyzed using a TOC analyzer (multi N/C 3100, Analytik Jena, Germany). Soil microbial biomass carbon (MBC) and microbial biomass nitrogen (MBN) were estimated using a chloroform fumigation extraction method (Brookes et al., 1985). Soil NH_4_^+^ and NO_3_^−^ concentrations were determined by extraction with 2 M KCl solution followed by colorimetric analysis on a FIAstar 5,000 Analyzer (FIAstar 5,000 Analyzer, Foss Tecator, Hillerød, Denmark). Soil pH was determined in a 1:2.5 soil: water solution (w/v).

### 2.4 DNA extraction, polymerase chain reaction (PCR) amplification, and purification

Soil DNA was extracted from a 0.3 g soil sample using the DNeasy® PowerSoil® Kit (Qiagen, Hilden, Germany) following the manufacturer’s instructions. The universal primers used for prokaryotic 16S rDNA amplification were 515F (5′-GTG CCA GCM GCC GCG GTA A-3′) (Caporaso et al., 2011) and 806R (5′-GGA CTA CHV GGG TWT CTA AT-3′) (Walters et al., 2016). The universal primers used for fungal ITS rDNA amplification were ITS3-F (5’-GCATCGATGAAGAACGCAGC-3′) and ITS4-R (5’-TCCTCCGCTTATTGATATGC-3′) (White et al., 1990). The PCR was carried out in 50 μL PCR reaction volumes, containing 15 μL of NEB Next Ultra II Q5 Mix (NEB, New Ipswich, MA, United States), 3 μL of the forward primer (10 μM), 3 μL of the reverse primer (10 μM), 1 μL of template DNA, and 8 μL of nuclease-free water. The thermal-cycling conditions were 95°C initial denaturation for 30 s, followed by 30 cycles of denaturation at 95°C for 5 min, annealing at 56°C for 30 s; extension at 72°C for 40 s, and a final extension at 72°C for 10 min. The PCR products were purified using the GeneJET Gel Extraction Kit (Thermo Fisher Scientific Inc., Waltham, MA, USA). Illumina NovaSeq high-throughput sequencing was performed with a paired-end (2 × 250 bp) sequencing strategy (Magigene Co., Ltd., Guangdong, China).

### 2.5 Amplicon high-throughput sequencing and bioinformatics analyses

The paired-end raw sequences were spliced using USEARCH (v11), and the low-quality sequences and primers were removed following the UPARSE pipeline (Edgar, 2013). The UNOISE3 denoising algorithm was used to generate zOTU representative sequences, and the zOTUs with sequence numbers <9 were removed (Edgar, 2016). The zOTU table was generated by mapping the zOTU representative sequence with the merged sequences via the otutab script. The taxonomic information annotations of the prokaryotes and fungi were prepared based on the SILVA138 and UNITE8.2 databases in QIIME2 (Quast et al., 2012; Nilsson et al., 2018; Bolyen et al., 2019). Finally, 24,131 and 4,004 zOTUs were obtained for prokaryotes and fungi, respectively. The prokaryotic and fungal sequence numbers in each sample were rarefied to 71,712 and 65,914, respectively, for subsequent analysis.

The rrn copy number of each OTU was searched using the rrnDB database and estimated according to its closest relatives with a known rrn copy (Stoddard et al., 2015). Then, we calculated the abundance-weighted average rrn copy number for each soil sample (Wu et al., 2017). We calculated the product of the estimated rrn copy number and the relative abundance of each OTU and summed these values of all OTUs for each sample.

### 2.6 Soil extracellular enzyme activity assays

Estimates of microbial activities, including hydrolases (α-glucosidase [AG], β-glucosidase [BG], and β-D-cellobiosidase [CB]) and oxidases (peroxidase [PEO] and polyphenol oxidase [PPO]) were based on the maximum potential activity of extracellular enzymes (Guan, 1986; Wallenstein et al., 2009).

The soil suspensions to measure the hydrolases were prepared by homogenizing a 1.0 g soil sample in 100 mL of 50 mmol L^−1^ sodium acetate buffer. Then, a mixture of soil homogenate, methylumbelliferyl (MUB), and a MUB-linked substrate was placed in a black polystyrene 96-well microplate and incubated in the dark for 4 h at 25°C. The hydrolytic enzyme activities were expressed as nmol g^−1^ h^−1^. The soil suspensions for oxidases were prepared by homogenizing a 1.0 g soil sample in 10 mL of 1% pyrogallol solution. The mixture was placed in an incubator after shock and cultured at 30°C for 2 h. The oxidative enzyme activities were expressed as mg g^−1^ h^−1^. The LCI was calculated as the ratio of lnPPO to the sum of lnPPO and lnBG (Duan et al., 2023). The units of the BG and PPO activities were converted to nmol g^−1^ MBC h^−1^ before calculating the LCI.

### 2.7 Statistical analyses

All statistical analyses were performed in R version 4.1.3 (R Development Core Team, 2022). Repeated-measurement analysis of variance was employed to examine the effects of drought, added N, and date on seasonal Rs and Rh using linear mixed-effect models and the R package nlme. The drought and nitrogen-added treatments were set as fixed effects, block was set as a random effect, and a corAR1type time-autocorrelated covariance matrix was used to avoid violating the assumption of sphericity for repeated measurement of Rs and Rh data. To examine the effects of drought and added N on soil properties, seasonal mean Rs and Rh, rrn copy number, and soil extracellular enzyme activities, we used linear mixed-effect models with the lme4 and lmerTest packages, setting drought and the nitrogen addition treatments as fixed effects and block as the random effect (Bates et al., 2015; Kuznetsova et al., 2017; Pinheiro et al., 2022). Multiple comparisons followed by linear mixed-effect models were performed with the R package lsmeans (Lenth, 2016). The standardized regression coefficient and marginal *R*^2^ were utilized to assess the effect size of fixed factors on Rh (Nakagawa and Schielzeth, 2013). The marginal *R*^2^ was calculated using the partR2 package (Stoffel et al., 2021). Pearson’s correlation coefficients between factors were examined and visualized using the R package ggcor.

Principal coordinates analysis (PCoA) and permutational multivariate analysis of variance (PERMANOVA) were performed using the vegan package (Oksanen et al., 2018) to reveal the effects of drought and added nitrogen on soil prokaryotic and fungal community composition. All community composition distances were calculated based on Bray-Curtis dissimilarities. Actinobacteriota, Acidobacteriota, and Chloroflexi were classified as the K-selected (oligotrophic-associated) bacterial phyla, and Proteobacteria, Bacteroidota, and Firmicutes were the r-selected (copiotrophic-associated) bacterial phyla (Phung et al., 2004; Fierer et al., 2007; Nemergut et al., 2010; Francioli et al., 2016). Basidiomycota was classified as a K-selected fungal phylum, and Ascomycota and Mortierellomycota were as r-selected fungal phyla (Yao F. et al., 2017; Wu et al., 2021). The bacterial or fungal phyla ratios of K- to r-strategists were calculated based on the relative abundance. The responses of the relative abundance of the bacterial and fungal lineages (from phylum to class) to drought were determined using the linear discriminant analysis effect size (LEfSe) method (Segata et al., 2011). The LEfSe was performed using the online Huttenhower Galaxy server (huttenhower.sph.harvard.edu/galaxy) with a setting LDA score > 4.0.

A structural equation model (SEM) was used with the R package piecewiseSEM (Lefcheck, 2016) to examine the causal pathways by which drought and adding N affected Rh. Based on our knowledge of the effects of drought and added N on Rh, we developed an *a priori* model to allow a hypothesized causal interpretation of the linkages between SWC, DOC, the LCI, prokaryotic community composition, fungal community composition, the abundance-weighted rrn copy number, the ratio of K- to r-selected bacteria (B_K:r_), the ratio of K- to r-selected fungi (F_K:r_), soil hydrolase activities, soil oxidases activities, and Rh. Prokaryotic and fungal community composition was represented by PC1 from the Bray-Curtis distance-based principal coordinate analysis. Soil hydrolase activities were calculated as the sum of the activities of AG, BG, and CB, and soil oxidase activities were calculated as the sum of PEO and PPO activities.

## 3 Results

### 3.1 Soil properties

After the drought treatments, SWC decreased significantly from 33.02 to 20.89% (*p* < 0.001), while ST increased significantly from 13.65°C to 14.53°C (*p* < 0.001; Supplementary Figure S1 and Supplementary Tables S1, S2). DOC increased significantly under drought and added N (*p* < 0.001; Supplementary Figure S1 and Supplementary Tables S1, S2). MBC did not change statistically under the drought and added N treatments (Supplementary Figure S1 and Supplementary Table S1). MBN decreased significantly under drought (*p* = 0.0024), and increased under added N (*p* < 0.001; Supplementary Figure S1 and Supplementary Tables S1, S2). Soil inorganic N concentration, NH_4_^+^, and NO_3_^−^, increased under the added N treatment (*p* = 0.0003, *p* < 0.001; Supplementary Figure S1 and Supplementary Tables S1, S2). Soil pH decreased significantly from 5.68 to 5.65 by 0.03 under the added N treatment (*p* = 0.00081; Supplementary Figure S1 and Supplementary Tables S1, S2).

### 3.2 Soil respiration and heterotrophic respiration

**Figure 1:**
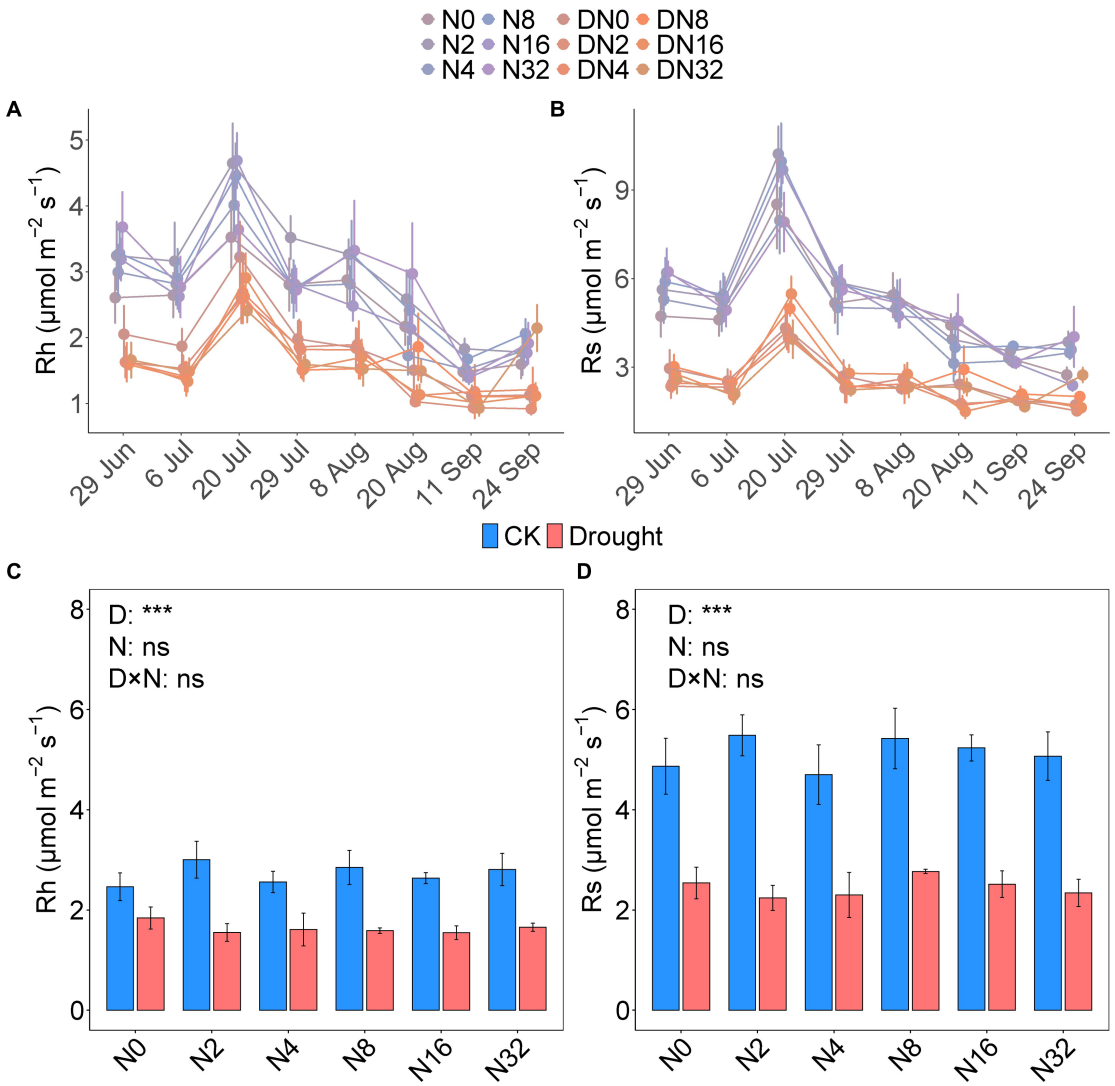
The seasonal dynamics of heterotrophic respiration (Rh, **A**) and soil respiration (Rs, **B**). The mean seasonal values of Rh **(C)** and Rs **(D)** under the different treatments. Data are mean ± S.E. (n = 4).

**Figure 2:**
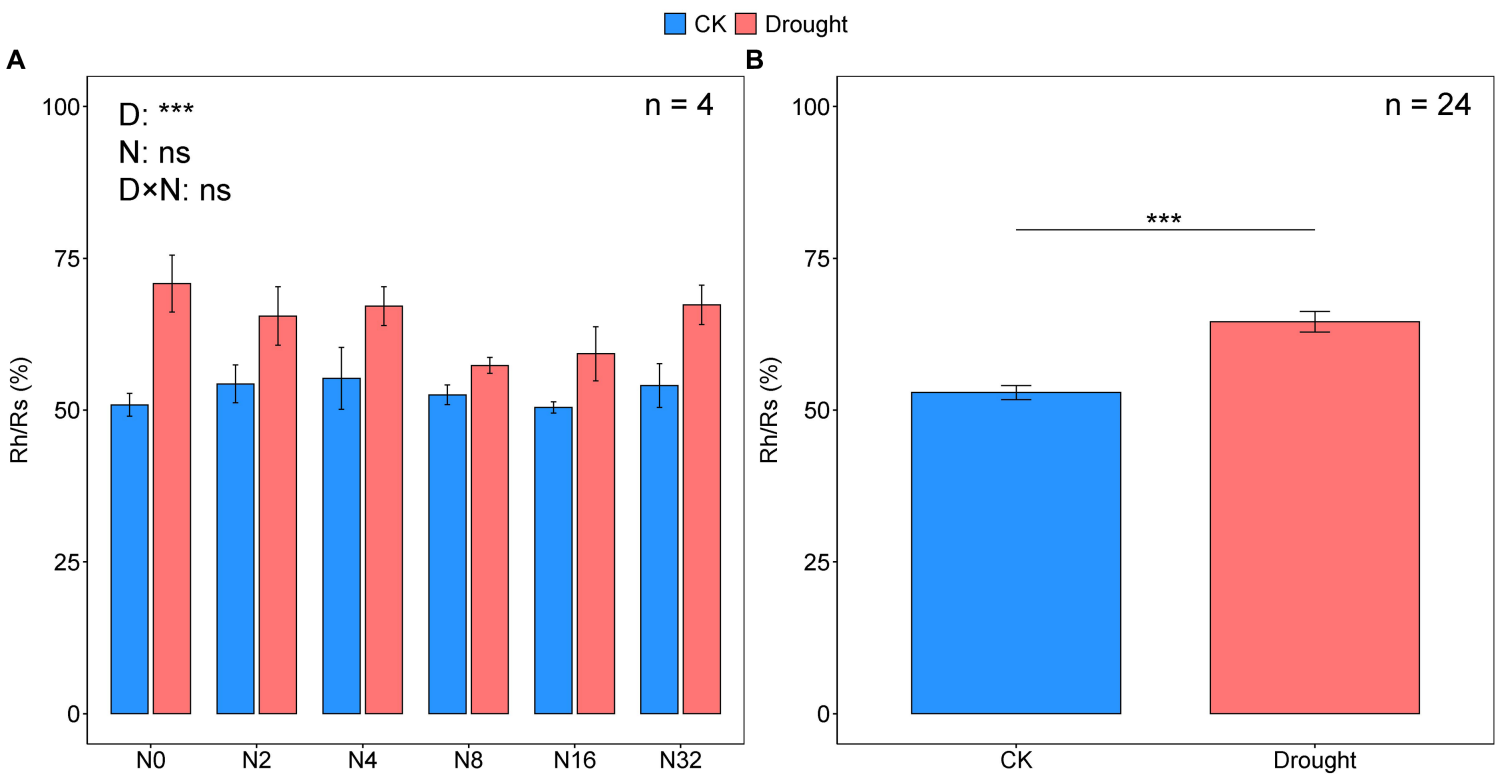
The ratio of heterotrophic respiration (Rh) to soil respiration (Rs) under the different treatments **(A)**, with or without the drought treatment **(B)**.

The temporal dynamics of soil respiration were consistent with the heterotrophic components, and all maximum rates occurred in July (Figures 1A,B; Table 1). Drought significantly decreased the mean growing season Rh and Rs values by 40.07 and 52.24%, respectively (*p* < 0.001, *p* < 0.001; Figures 1C,D; Supplementary Tables S1, S2). While adding N had no effect on the mean growing season Rh and Rs values (Figures 1C,D; Supplementary Tables S1, S2). The mean Rh/Rs ratio increased significantly over the growing season by 22.04% under drought (*p* < 0.001; Figure 2B; Supplementary Tables S1, S2), but did not change significantly under the N addition gradient (Figure 2A; Supplementary Table S1). Notably, the effects of adding N on Rs and Rh were not significant for the seasonal dynamics and the mean value (Figures 1A,B; Supplementary Table S1). Drought significantly increased the Rh to Rs ratio from 52.91 ± 1.17 to 64.57 ± 1.70 (*p* < 0.001), whereas adding N did not affect the Rh to Rs ratio (Figure 2A; Supplementary Tables S1, S2).

**Table 1:** Results from linear mixed models for the effects of date, drought, added nitrogen, and their interactions on Rh and Rs.

	Rh		Rs	
	*F*	*p*	*F*	*p*
Date	81.24	**<0.001** ^***^	135.16	**<0.001** ^***^
Drought	60.41	**<0.001** ^***^	126.63	**<0.001** ^***^
Nitrogen	0.21	0.95	0.47	0.79
Drought× Nitrogen	0.69	0.64	0.33	0.89
Drought× Date	6.36	**<0.001** ^***^	16.64	**<0.001** ^***^
Nitrogen× Date	1.38	0.085	1.49	**0.045** ^*^
Drought× Nitrogen× Date	0.89	0.64	1.03	0.43

Bold values represent statistically significant. ^*^0.01 < *p* ≤ 0.05; ^**^0.001 < *p* ≤ 0.01; ^***^p ≤ 0.001.

### 3.3 Soil microbial community composition

**Figure 3:**
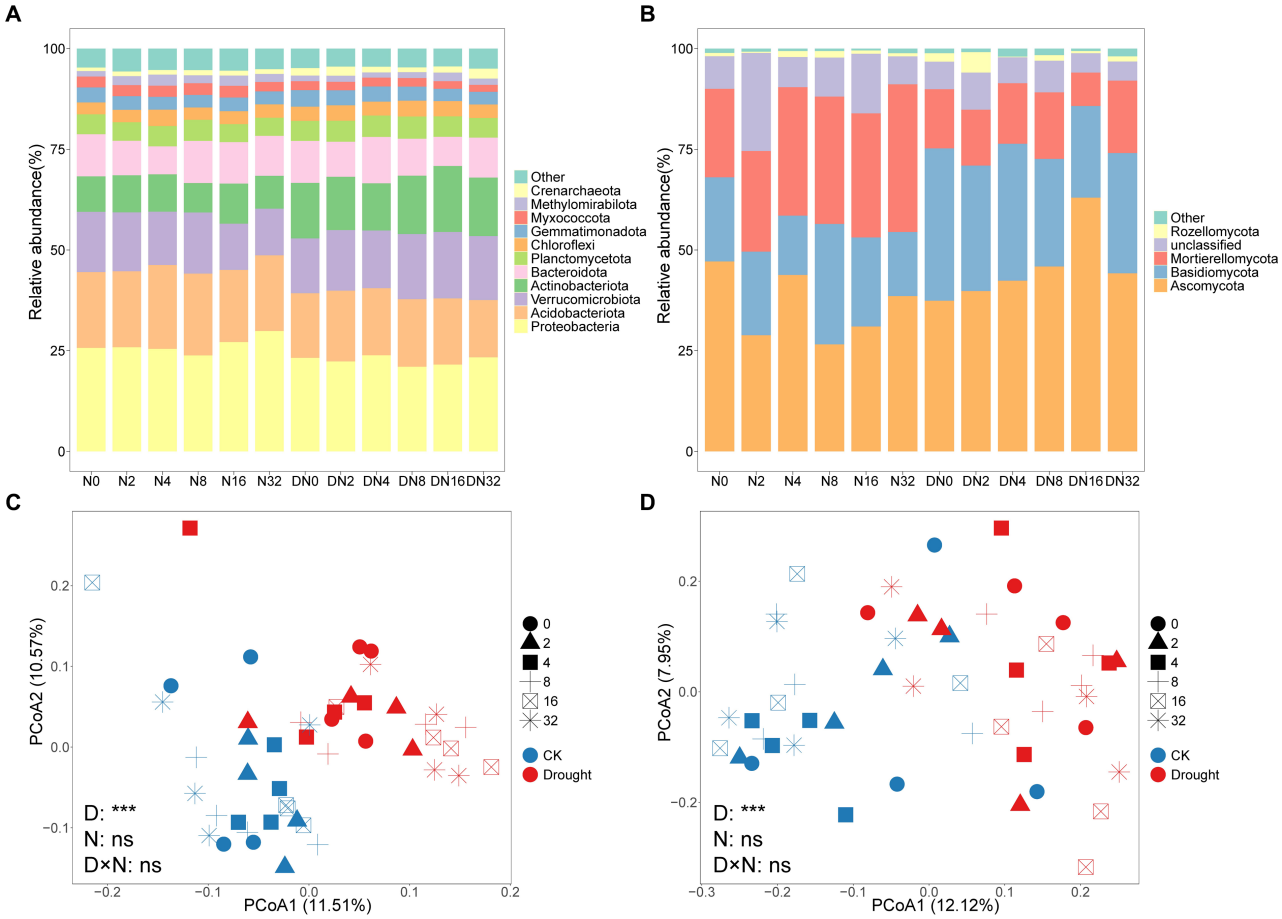
Relative abundance of the dominant **(A)** prokaryotic and **(B)** fungal groups at the phylum level under the different treatments. Principal coordinates analysis (PCoA) based on the **(C)** prokaryotic and **(D)** fungal communities.

As shown in Figure 3A, the dominant prokaryotic phylum was Proteobacteria, followed by Acidobacteria, Verrucomicrobiota, Actinobacteriota, and Bacteroidota. As shown in Figure 3B, the dominant fungal phylum was Ascomycota, followed by Basidiomycota and Mortierellomycota.

**Figure 4:**
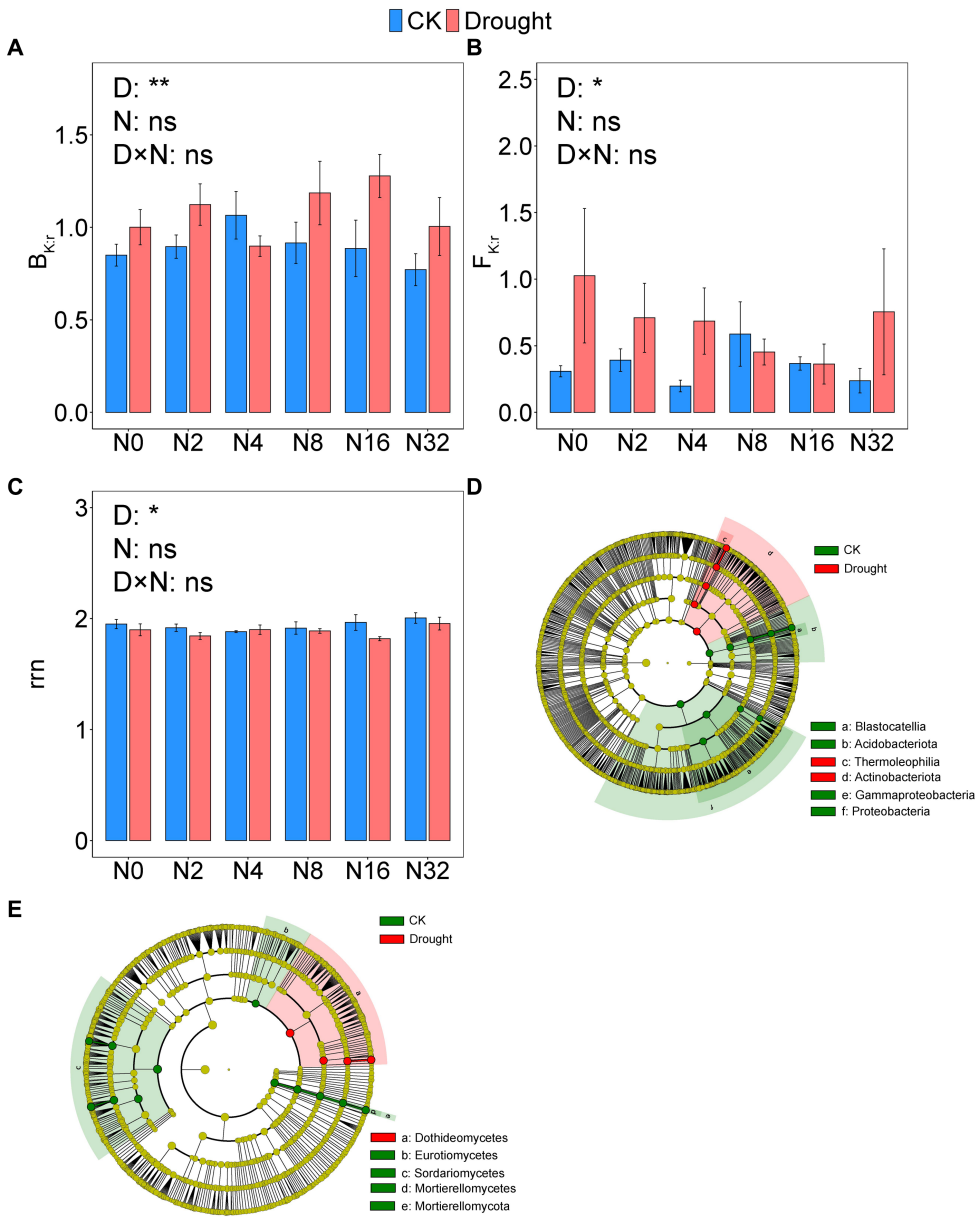
The ratio of K- to r-strategist **(A)** bacterial phyla (B_K:r_) and **(B)** fungal phyla (F_K:r_); **(C)** abundance-weighted average rRNA operon (rrn) copy numbers and linear discriminant analysis effect size (LEfSe) of the **(D)** bacteria and **(E)** fungi. Data are mean ± S.E. (*n* = 4).

Drought significantly changed prokaryotic (*p* = 0.0001, PERMANOVA) and fungal (*p* = 0.0001, PERMANOVA) community composition, while adding N had no effect (Supplementary Table S3). Drought increased B_K:r_ from 0.90 ± 0.042 to 1.08 ± 0.052 (*p* = 0.0051), and F_K:r_ from 0.35 ± 0.049 to 0.67 ± 0.126 (*p* = 0.014), respectively (Figures 4A,B; Supplementary Tables S1, S2). The rrn value decreased significantly from 1.94 ± 0.019 to 1.88 ± 0.017 under drought (*p* = 0.035; Figures 4A–C; Supplementary Tables S1, S2). However, adding N did not significantly change prokaryotic or fungal community composition or rrn (Figures 4A–C; Supplementary Table S1).

As revealed by the LEfSe analysis of bacteria at the phylum level, the relative abundance of Acidobacteriota and Proteobacteria decreased under drought, while the relative abundance of Actinobacteriota increased. The relative abundance of Thermoleophilia increased under drought, while the relative abundance of Blastocatellia, and Gammaproteobacteria decreased (Figure 4D). The LEfSe analysis of fungi at the phylum level revealed that drought significantly reduced the relative abundance of Mortierellomycota. Drought increased the relative abundance of Dothideomycetes but decreased that of Eurotiomycetes, Sordariomycetes, and Mortierellomycetes (Figure 4E).

### 3.4 Soil extracellular enzyme activities

**Figure 5:**
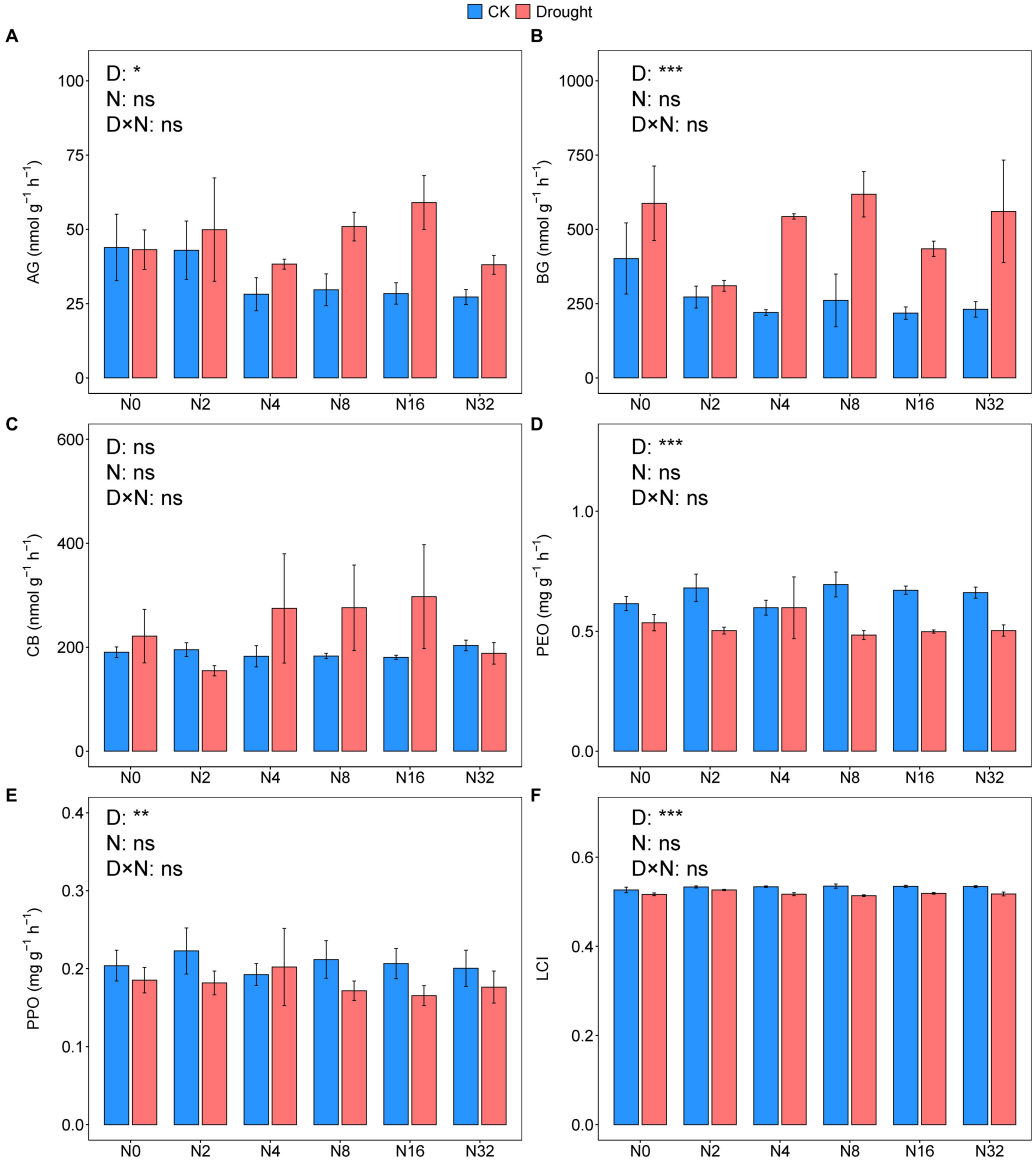
Enzyme activities of **(A)** α-glucosidase, **(B)** β-glucosidase, **(C)** β-D-cellobiosidase, **(D)** peroxidase, **(E)** polyphenol oxidase, and **(F)** the lignocellulose index under the different treatments. Data are mean ± S.E. (*n* = 4).

Drought significantly increased the activities of AG and BG by 39.42 and 90.53%, respectively (*p* = 0.0072, *p* < 0.001, *p* < 0.014; Figures 5A,B; Supplemenatry Tables S1, S2). However, the activities of PEO and PPO decreased by 20 and 14.29%, respectively under drought (*p* < 0.001, *p* = 0.0026; Figures 5D,E; Supplemenatry Tables S1, S2). Adding N did not significantly change any of the enzyme activities (Figure 5; Supplemenatry Table S1). Drought significantly decreased the LCI from 0.53 to 0.52 (*p* < 0.001; Figure 5F, Supplemenatry Table S2).

### 3.5 Correlations between the environmental factors and heterotrophic respiration

Pearson’s correlations showed that Rh and Rs were significantly positively correlated with SWC, MBN, rrn, PEO, PPO, and the LCI (Supplementary Figure S3), and significantly negatively correlated with DOC, 16SPC1, ITSPC1, and BG (Supplemenatry Figure S3). Rh was significantly positively correlated with MBC, while Rs were significantly negatively correlated with AG and B_K:r_ (Supplementary Figure S3).

**Figure 6:**
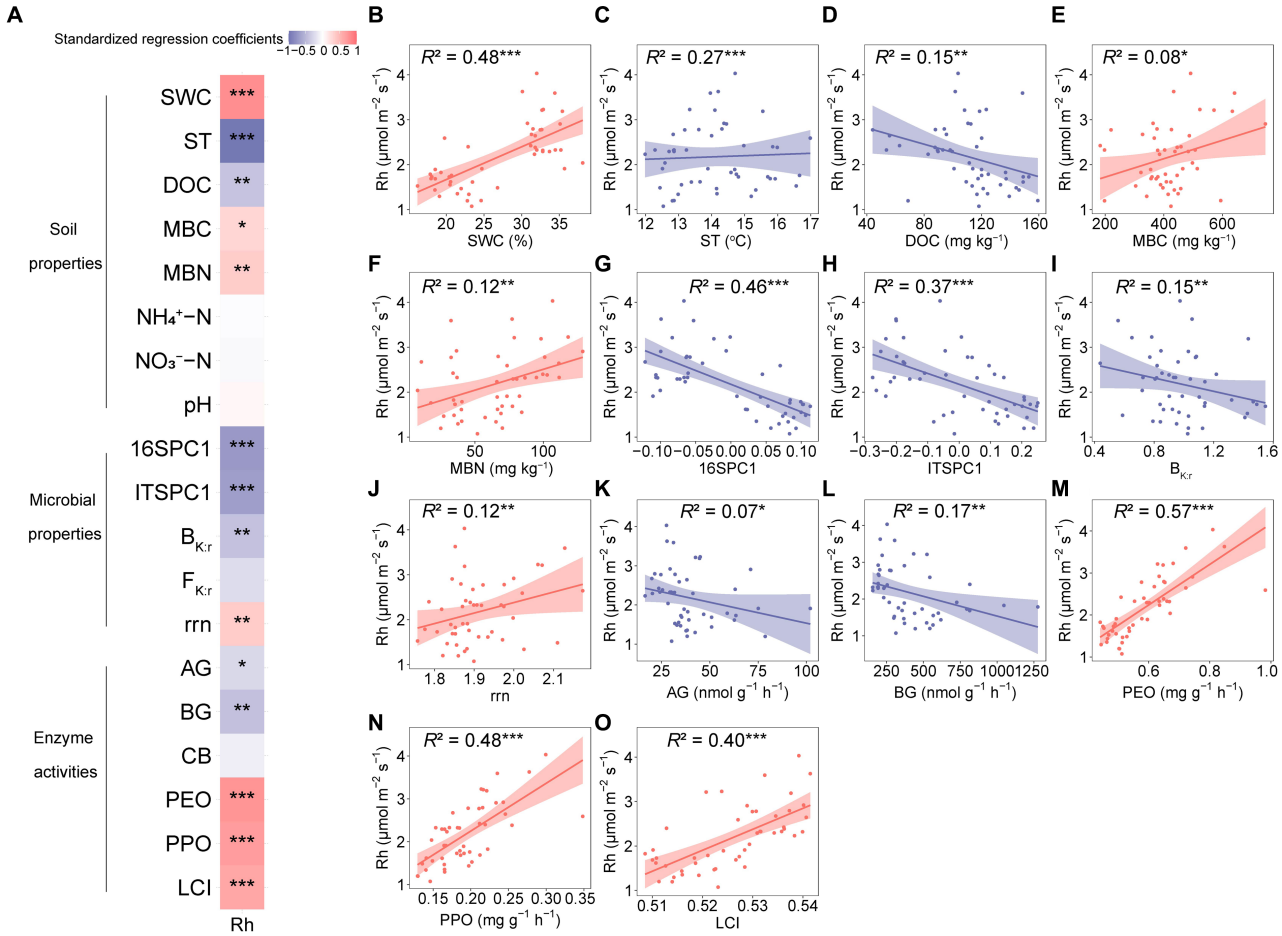
Relationships between heterotrophic respiration (Rh) and biotic and abiotic factors. **(A)** Standardized regression coefficients for the biotic and abiotic factors with Rh. **(B–O)** Relationships between Rh and soil properties, microbial properties, and enzyme activities. The solid lines are regression curves based on linear mixed effect models, and the shaded areas represent the 95% confidence intervals. SWC, soil water content; ST, soil temperature; DOC, dissolved organic carbon; MBC, microbial biomass carbon; MBN, microbial biomass nitrogen; NH_4_^+^-N, ammonium-nitrogen; NO_3_^−^-N, nitrate-nitrogen; 16SPC1, prokaryotic community composition; ITSPC1, fungal community composition; B_K:r_, the ratio of K- to r-strategists of bacterial phyla; F_K:r_, the ratio of K- to r-strategists of fungal phyla; rrn, abundance-weighted average rRNA operon copy number; AG, α-glucosidase; BG, β-glucosidase; CB, β-D-cellobiosidase; PEO, peroxidase; PPO, polyphenol oxidase; LCI, the lignocellulose index; Rh, seasonal mean heterotrophic respiration. ^*^0.01 < *p* ≤ 0.05; ^**^0.001 < *p* ≤ 0.01; ^***^p ≤ 0.001.

We analyzed the relationships between potential drivers (i.e., soil properties, microbial properties, and enzyme activities) and Rh to explore the controls of Rh. As a result, Rh was significantly associated with the factors (Figure 6A). Briefly, for soil properties, ST and DOC significantly attenuated Rh (Figures 6C,D). In contrast, Rh was significantly facilitated by the increases in SWC, MBC, and MBN (Figures 6B,E,F). While Rh was not correlated with NH_4_^+^-N, NO_3_^−^-N, or pH (Figure 6A). For microbial properties, the prokaryotic community composition, fungal community composition, and B_K:r_ were negatively correlated with Rh (Figures 6G,H,I), while rrn was positively correlated with Rh (Figure 6J). For enzyme activities, Rh decreased with AG and BG but increased with PEO, PPO, and LCI (Figures 6K–O).

**Figure 7:**
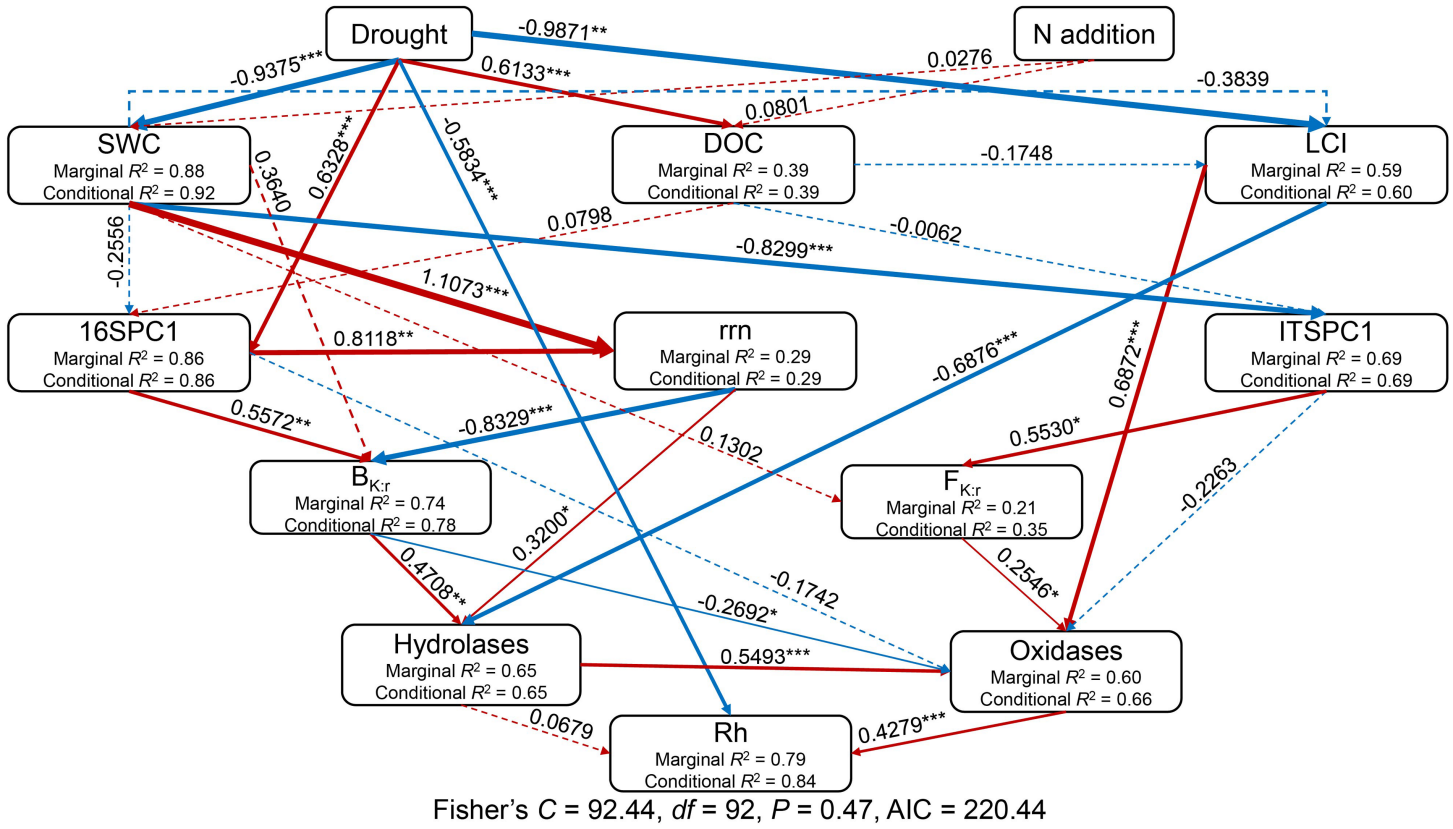
Structural equation model considering the plausible pathways through which drought and added nitrogen affect heterotrophic respiration (Rh). Before the SEM analysis, prokaryotic and fungal OTU tables were subject to principal coordinates analysis (PCoA) to generate PC1 representing prokaryotic and fungal community composition. The activities of AG, BG, and CB were summed to represent the hydrolases, while the activities of PEO and PPO were summed to represent the oxidases. Red and blue arrows represent positive and negative pathways, respectively, while solid and dashed arrows indicate significant and nonsignificant pathways, respectively. Numbers at arrows are standardized path coefficients and arrow width is proportional to the strength of the relationship. ^*^0.01 < *p* ≤ 0.05; ^**^0.001 < *p* ≤ 0.01; ^***^p ≤ 0.001. Conditional *R*^2^ and marginal *R*^2^ values near response variables indicate the proportion of variation explained by response variables with and without random effect. The final results of model fitting: Fisher’s *C* = 92.44, *p* = 0.47, *df* = 92, *n* = 48, Akaike information criteria (AIC) = 220.44.

**Figure 8:**
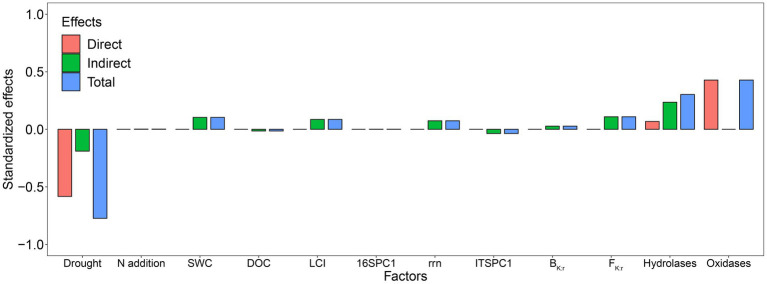
Standardized direct, indirect, and total effect sizes of factors on Rh.

The SEM explained 84% of the variation in Rh (Figure 7). Standardized total effects from the SEM showed that drought had a significant negative effect on Rh while adding N did not (Figures 7, 8). Except for drought, oxidases were the most significant factor affecting Rh (Figures 7, 8), in which oxidases exerted a positive effect. The standardized direct effect sizes of drought, hydrolases, and oxidases on Rh were − 0.5834, 0.0679, and 0.4279, respectively (Figure 8), but only the drought and oxidase pathways were significant (Figure 7). Drought, SWC, LCI, rrn, ITSPC1, B_K:r_, F_K:r_, and hydrolases were important factors that exerted indirect effects on Rh (Figures 7, 8). LCI, F_K:r_, and hydrolases contributed to increased oxidases, the second most important factor that affected Rh, while B_K:r_ contributed to decreasing oxidases.

## 4 Discussion

### 4.1 The shift in oxidase activity induced by the microbial life history strategy-mediated Rh response to drought and added nitrogen

Previous studies have shown that drought and adding N change the microbial community composition (Allison et al., 2007; Treseder, 2008; Gao et al., 2021). However, our results show that only drought altered the microbial community composition (Figures 3C,D), which partially supported our hypothesis 1. The results of a study in semi-arid grasslands showed that reducing precipitation increases oligotrophs and decreased copiotrophs, which was consistent with our B_K:r_ results (Li et al., 2022). A lower proportion of r-selection and average potential growth rate under drought has been suggested by a lower abundance-weighted average rrn copy number (Roller et al., 2016). Consistent with a previous study (Bu et al., 2018), the abundance of Gammaproteobacteria, which has often been associated with copiotrophic bacteria (Kurm et al., 2017), was suppressed by drought (Figure 4C). Here, the increased B_K:r_, F_K:r_, and decreased rrn support that drought favors oligotrophic taxa. Water affects microbial dynamics as a transport medium (Tecon and Or, 2017). Our drought treatments significantly reduced SWC (Supplementary Tables S1, S2) and, therefore, decreased diffusion pathways of dissolved nutrients (Tecon and Or, 2017). The breakdown of hydrological links is thought to be the main reason why drought affects soil community composition (Carson et al., 2010). Pearson’s correlation analysis revealed that SWC was significantly correlated with B_K:r_, F_K:r_, and rrn (Supplementary Figure S3). Thus, the drought-induced decrease in SWC drove the microbes to shift to an oligotroph-dominated community.

The results in extracellular enzyme activities did not accord with our hypothesis 2. In this study, drought increased hydrolase activities, including AG and BG. Previous studies have reported that the potential activities of AG and BG increase under dry conditions, indicating a decrease in hydrolase turnover (Alster et al., 2013; Ochoa-Hueso et al., 2020). Furthermore, partly consistent with our third hypothesis, only drought decreased Rh in this study (Figure 1C), which agrees with some previous research on terrestrial ecosystems (Zhou L. Y. et al., 2016; Zhou X. et al., 2016; Veach and Zeglin, 2020; Zheng et al., 2021). Our SEM showed that drought reduced oxidases via the LCI, B_K:r_, F_K:r_, and hydrolases and subsequently, Rh (Figure 7). Drought affected fungal community composition by decreasing SWC and the LCI, then increasing F_K:r_ and decreasing oxidase activities. The B_K:r_ and F_K:r_ values increased, and the rrn value decreased in this study, indicating that drought shifted the microbial community from r-strategist-dominated to K-strategist-dominated (Figures 4A–C) (Roller et al., 2016; Duan et al., 2023). K-strategists are more associated with oxidases than r-strategists and effectively utilize recalcitrant C, including lignin (Chen et al., 2022; Morrissey et al., 2023). Our SEM showed that the increase in B_K:r_ and the decrease in rrn promoted hydrolase activity. However, hydrolases had no significant effect on Rh (Figure 7). Soil hydrolases and oxidases are related to labile and recalcitrant C, respectively (Sinsabaugh et al., 2008; Burns et al., 2013). Here, drought reduced the LCI (Figure 6F), indicating that the C substrate was more vulnerable to decomposition (Moorhead et al., 2013). According to the optimization of the cost/benefit ratio (Allison et al., 2011), more labile soil C substrate should increase the activities of hydrolase, and decrease that of oxidase. Microbial community structure may be less important for the turnover of more labile C because a broad phylogeny of taxa is capable of metabolizing simpler compounds (Berg and McClaugherty, 2014). In that case, the rate-limiting variables for Rh may depend more on oxidase than hydrolase activity of the microbial community even if the microbial life history strategy shifts. The r-strategist-dominated soils generally have a higher respiration rate than K-strategist-dominated soils, which may decrease Rh by reducing oxidase activities (Bailey et al., 2002; Six et al., 2006; Fierer et al., 2007; Waring et al., 2013; Malik et al., 2016). Oxidase genes were identified in γ-Proteobacteria, indicating it could be a potential oxidase producer (Tian et al., 2014). In this study, the relative abundance of γ-Proteobacteria decreased under drought (Figure 4D), causing the reduction of oxidase activities. According to the LEfSe, Mortierellomycota contributed to the change in the F_K:r_ value at the phylum level (Figure 4E). Moreover, members of Mortierellomycota are sensitive to reduced precipitation (Han et al., 2024). A previous study found that the relative abundance of Mortierellomycota is lower in dry ecosystems (Tedersoo et al., 2014). The phyla Mortierellomycota mostly includes saprotrophs in the soil (James et al., 2006; Tedersoo et al., 2018). Saprophytic fungi perform the initial steps in the decomposition of cellulose, lignin, and other complex macromolecules (Gessner et al., 2010; Berg and McClaugherty, 2014). Mortierellomycota is involved in the decomposition of recalcitrant C (Větrovský and Baldrian, 2013; Fang et al., 2018; Shi et al., 2020), this may be the reason why F_K:r_ increases oxidase activities. From the correlation analysis and structural equation model (Figures 7, 8), the bacterial community composition shift exerted a greater effect on oxidase activities than fungal community composition shift, which ultimately led to the decline of oxidase activity, and thus a decrease in Rh.

### 4.2 Uncertainties

The estimate of Rh in this study may have some limitations. First, the Rh value may have been overestimated, because the trenched subplots allowed root ingrowth underneath the collar (>0.6 m depth) into the subplots (Sayer and Tanner, 2010). Second, trenched subplots may exhibit a different microbial community composition from non-trenched subplots, which would change the Rh value (Chen et al., 2016). Third, long-term collar deployment leads to bias in soil respiration measurements, which contributes to higher soil bulk density and lower microbial biomass; inside long-term collars can underestimate Rh (Ma et al., 2023). Finally, the conclusions were drawn from Rh during the growing season. It is necessary to consider the seasonal pattern of Rh in response to drought and added N, including the growing and nongrowing seasons. In addition, we also assayed the potential activities of soil extracellular enzymes. An assay of potential enzyme activities usually provides an unlimited and relatively simple soluble substrate and is usually performed at a constant temperature, which is inconsistent with reality and may result in a misestimate of enzyme activity (Wallenstein and Weintraub, 2008).

In this study, adding N had no significant effect on Rh. Adding N has been reported to reduce Rh by decreasing microbial biomass (Treseder, 2008; Liu and Greaver, 2010). Here, MBC did not change significantly after adding N (Supplementary Table S1). Previous global meta-analysis revealed that MBC decreases with increasing experimental duration, indicating that the negative effects of adding N on microbes become more pronounced over time (Zhang et al., 2018). We predict that adding N will decrease Rh because soil microbes suffer progressive inhibition and continue to decrease in the long term. However, drought increases the availability of nitrogen, which can harm phenols, and reductions in phenols can increase Rh. Therefore, experiments on the effects of long-term drought and added nitrogen on Rh are full of uncertainties and should be continuously conducted.

## 5 Conclusion

In summary, this study revealed the regulatory mechanisms underlying the Rh responses to drought and adding N by integrating soil properties, microbial life history strategies, and extracellular enzyme activities. Our findings show that drought decreased Rh primarily by inhibiting oxidase activities, which is induced by bacterial shifts from the r-strategy to the K-strategy. The changes in extracellular enzymes highlight the importance of the dynamics of the ratio of K- to r-selected in bacterial and fungal communities in regulating Rh. However, adding N did not affect Rh, which emphasizes the necessity for long-term observations. Therefore, the dynamic of bacterial and fungal life history strategy should be fully considered for a better understanding of the responses of terrestrial ecosystems to future climate change scenarios.

## Data availability statement

The data presented in the study are deposited in the NCBI BioProject under accession number (PRJNA1012514). The datasets presented in this study can be found in online repositories. The names of the repository/repositories and accession number(s) can be found at: https://www.ncbi.nlm.nih.gov/, PRJNA1012514.

## Author contributions

WZ: Writing – original draft. YL: Conceptualization, Writing – review & editing. XK: Writing – review & editing. LY: Writing – review & editing. XZ: Writing – review & editing. ZY: Writing – review & editing. KZ: Writing – review & editing. AY: Writing – review & editing. YN: Writing – review & editing. XY: Writing – review & editing. HW: Writing – review & editing. MA: Writing – review & editing. RC: Writing – original draft.
