# Supplementary material for: Changes in soil oxidase activity induced by microbial life history strategies mediate the soil heterotrophic respiration response to drought and nitrogen enrichment
Source: Front Microbiol. 2024 Mar 15;15:1375300. doi: 10.3389/fmicb.2024.1375300 (PMC10978626; doi:10.3389/fmicb.2024.1375300)
Supplement: Supplementary file 1 [file Data_Sheet_1.docx]

**Table S1** Results from the linear mixed model for the effects of drought and added nitrogen and their interaction on soil properties, microbial properties, enzyme activities, Rh, Rs, and the Rh to Rs ratio. * 0.01 < *P* ≤ 0.05; ** 0.001 < *P* ≤ 0.01; *** *P* ≤ 0.001

|  | Drought |  | Nitrogen |  | Drought× Nitrogen | |
| --- | --- | --- | --- | --- | --- | --- |
| Factor | *F* | *P* | *F* | *P* | *F* | *P* |
| SWC | 431.87 | **<0.001***** | 0.77 | 0.58 | 0.095 | 0.99 |
| ST | 29.23 | **<0.001***** | 1.47 | 0.23 | 0.26 | 0.93 |
| DOC | 36.25 | **<0.001***** | 3.35 | **0.014*** | 1.48 | 0.22 |
| MBC | 2.31 | 0.14 | 1.17 | 0.34 | 0.72 | 0.61 |
| MBN | 10.79 | **0.0024**** | 7.39 | **<0.001***** | 1.98 | 0.11 |
| NH_4_^+^-N | 2.33 | 0.14 | 6.19 | **0.0003***** | 0.66 | 0.65 |
| NO_3_^-^-N | 2.37 | 0.13 | 28.8 | **<0.001***** | 1.13 | 0.36 |
| pH | 0.32 | 0.57 | 5.55 | **0.00081***** | 0.79 | 0.57 |
| B_K:r_ | 9.02 | **0.0051**** | 0.96 | 0.46 | 1.58 | 0.19 |
| F_K:r_ | 6.79 | **0.014*** | 0.47 | 0.79 | 1.21 | 0.33 |
| rrn | 4.81 | **0.035*** | 1.42 | 0.24 | 0.78 | 0.57 |
| AG | 8.13 | **0.0072**** | 1.05 | 0.4 | 0.97 | 0.45 |
| BG | 24.28 | **<0.001***** | 0.99 | 0.44 | 1.05 | 0.41 |
| CB | 2.51 | 0.12 | 0.46 | 0.79 | 0.81 | 0.55 |
| PEO | 27.26 | **<0.001***** | 0.065 | 0.99 | 1.58 | 0.19 |
| PPO | 10.66 | **0.0026**** | 0.37 | 0.86 | 1.05 | 0.4 |
| LCI | 64.81 | **<0.001***** | 1.55 | 0.2 | 1.47 | 0.23 |
| Rh | 28.38 | **<0.001***** | 0.38 | 0.54 | 0.81 | 0.37 |
| Rs | 273.51 | **<0.001***** | 1.039 | 0.41 | 0.67 | 0.65 |
| Rh/Rs | 35.51 | **<0.001***** | 1.58 | 0.19 | 1.1 | 0.38 |

SWC: soil water content; ST: soil temperature; DOC: dissolved organic carbon; MBC: microbial biomass carbon; MBN: microbial biomass nitrogen; NH_4_^+^-N: ammonium-nitrogen; NO_3_^-^-N: nitrate-nitrogen; B_K:r_, the ratio of K- to r-strategists of bacterial phyla; F_K:r_, the ratio of K- to r-strategists of fungal phyla; rrn: abundance-weighted average rRNA operon copy number; AG: α-glucosidase; BG: β-glucosidase; CB: β-D-cellobiosidase; PEO: peroxidase; PPO: polyphenol oxidase; LCI: the lignocellulose index; Rh: seasonal mean heterotrophic respiration; Rs: seasonal mean soil respiration.

**Table S2** Mean ± standard error values for soil properties, microbial properties, enzyme activities, Rh, Rs, and the Rh to Rs ratio of the different treatments.

|  | CK | Drought | N0 | Nitrogen |
| --- | --- | --- | --- | --- |
| SWC (%) | 33.02±0.39 | 20.89±0.54 | 27.15±2.34 | 26.91±1.05 |
| ST (℃) | 13.65±0.21 | 14.53±0.28 | 14.18±0.47 | 14.07±0.21 |
| DOC (mg kg^-1^) | 94.72±4.78 | 127.19±3.90 | 114.55±7.05 | 110.24±4.44 |
| MBC (mg kg^-1^) | 444.60±27.57 | 396.70±14.93 | 434.06±22.42 | 417.97±18.61 |
| MBN (mg kg^-1^) | 74.22±7.12 | 53.86±4.39 | 83.14±9.35 | 60.22±4.75 |
| NH_4_^+^-N (mg kg^-1^) | 9.60±6.23 | 21.96±7.84 | 2.90±1.32 | 18.36±5.96 |
| NO_3_^-^-N (mg kg^-1^) | 30.17±5.23 | 35.27±4.17 | 19.17±4.36 | 35.43±3.77 |
| pH | 5.68±0.051 | 5.65±0.045 | 5.77±0.055 | 5.64±0.038 |
| B_K:r_ | 0.90±0.042 | 1.08±0.052 | 0.93±0.059 | 1.00±0.041 |
| F_K:r_ | 0.35±0.049 | 0.67±0.126 | 0.67±0.271 | 0.47±0.067 |
| rrn | 1.94±0.019 | 1.88±0.017 | 1.93±0.033 | 1.91±0.015 |
| AG (nmol g^-1^ h^-1^) | 33.38±2.96 | 46.54±3.55 | 43.51±6.04 | 39.25±2.74 |
| BG (nmol g^-1^ h^-1^) | 267.08±26.70 | 508.88±40.31 | 366.66±30.53 | 494.58±87.51 |
| CB (nmol g^-1^ h^-1^) | 189.29±4.60 | 235.51±28.09 | 206.03±24.96 | 213.67±16.75 |
| PEO (mg g^-1^ h^-1^) | 0.65±0.015 | 0.52±0.022 | 0.58±0.026 | 0.59±0.019 |
| PPO (mg g^-1^ h^-1^) | 0.21±0.008 | 0.18±0.009 | 0.20±0.012 | 0.19±0.007 |
| LCI | 0.53±0.001 | 0.52±0.001 | 0.52±0.004 | 0.53±0.002 |
| Rh (μmol m^-2^ s^-1^) | 2.72±0.11 | 1.63±0.072 | 2.15±0.20 | 2.18±0.12 |
| Rs (μmol m^-2^ s^-1^) | 5.13±0.19 | 2.45±0.11 | 3.70±0.53 | 3.81±0.25 |
| Rh/Rs (%) | 52.91±1.17 | 64.57±1.70 | 60.86±4.44 | 58.32±1.35 |

SWC: soil water content; ST: soil temperature; DOC: dissolved organic carbon; MBC: microbial biomass carbon; MBN: microbial biomass nitrogen; NH_4_^+^-N: ammonium nitrogen; NO_3_^-^-N: nitrate nitrogen; B_K:r_, the ratio of K- to r-strategists of bacterial phyla; F_K:r_, the ratio of K- to r-strategists of fungal phyla; rrn: abundance-weighted average rRNA operon copy number; AG: α-glucosidase; BG: β-glucosidase; CB: β-D-cellobiosidase; PEO: peroxidase; PPO: polyphenol oxidase; LCI, the lignocellulose index; Rh: seasonal mean heterotrophic respiration; Rs: seasonal mean soil respiration. CK: CK treatment. Drought: drought treatment. N0: treatments without nitrogen deposition. Nitrogen: treatments with nitrogen deposition.

**Table S3** Permutational multivariate analysis of variance (PERMANOVA) for prokaryotic and fungal composition. * 0.01 < *P* ≤ 0.05; ** 0.001 < *P* ≤ 0.01; *** *P* ≤ 0.001

|  | Drought |  | Nitrogen |  | Drought× Nitrogen | |
| --- | --- | --- | --- | --- | --- | --- |
|  | *F* | *P* | *F* | *P* | *F* | *P* |
| Prokaryotes | 5.07 | **0.0001***** | 0.99 | 0.51 | 1.04 | 0.35 |
| Fungi | 4.30 | **0.0001***** | 0.96 | 0.62 | 0.93 | 0.76 |


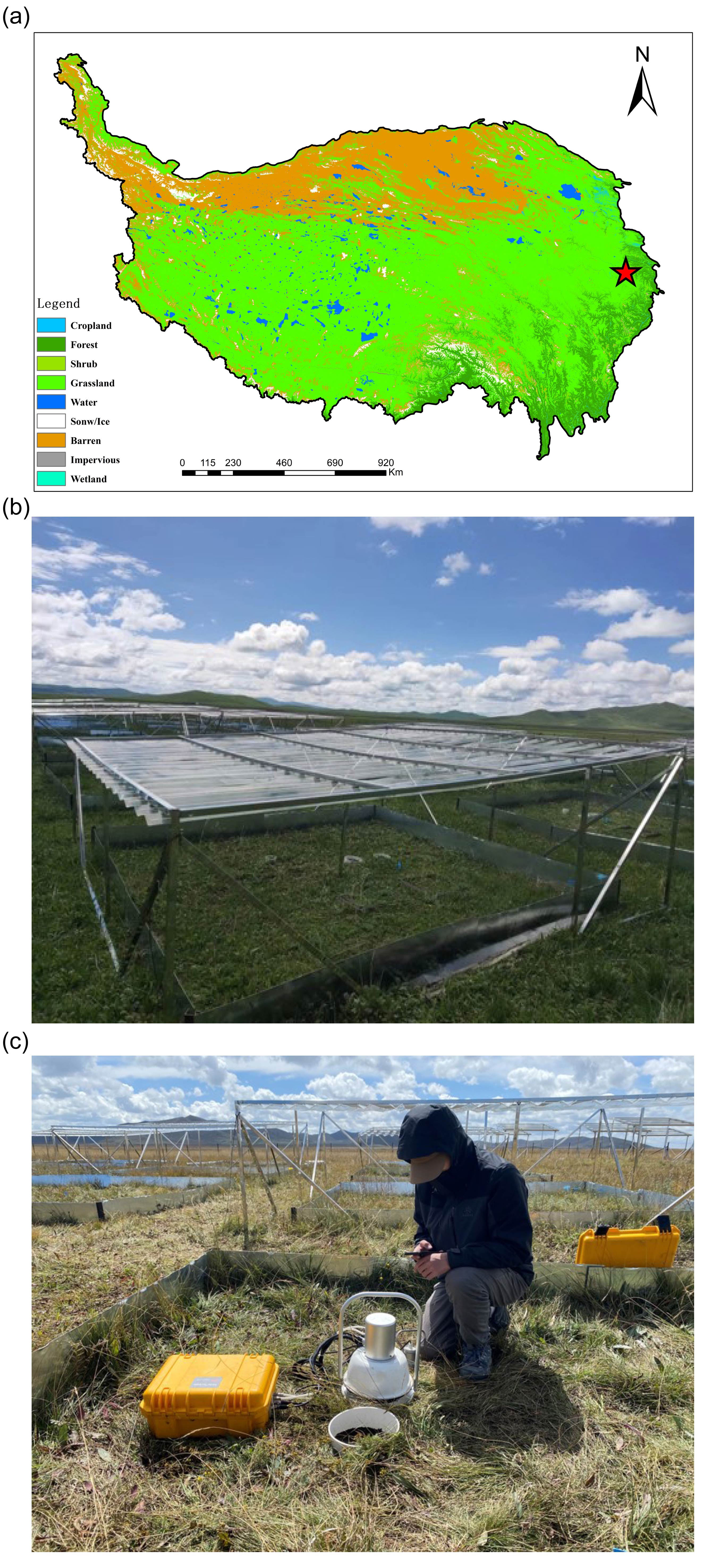


**Fig. S1** (a) The red star represents the location of the study site; (b) plot under drought treatment; (c) Rs and Rh measurements.


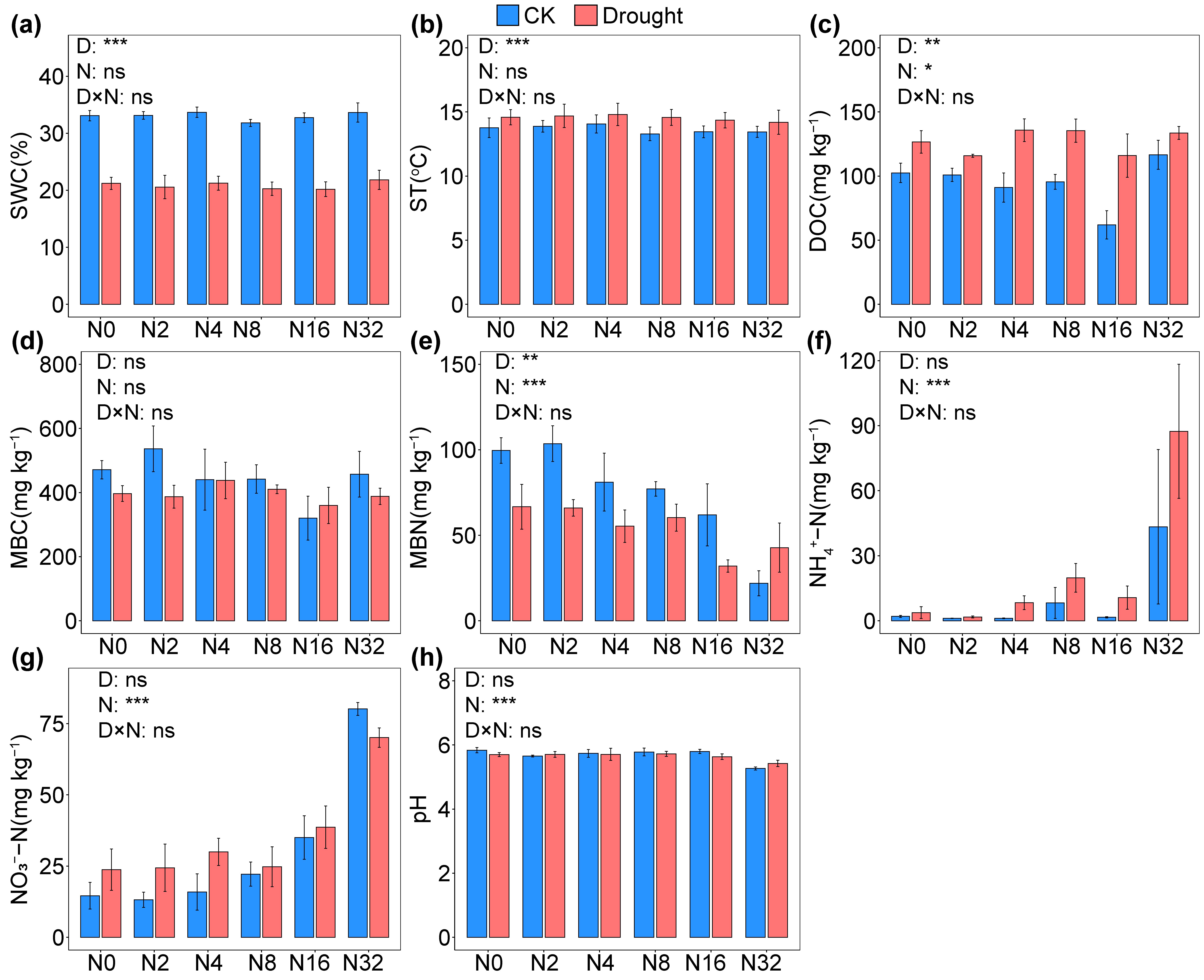


**Fig. S2** Soil properties. (a) soil water content; (b) soil temperature; (c) dissolved organic carbon; (d) microbial biomass carbon; (e) microbial biomass nitrogen; (f) ammonium-nitrogen; (g) nitrate-nitrogen; (h) pH.

**
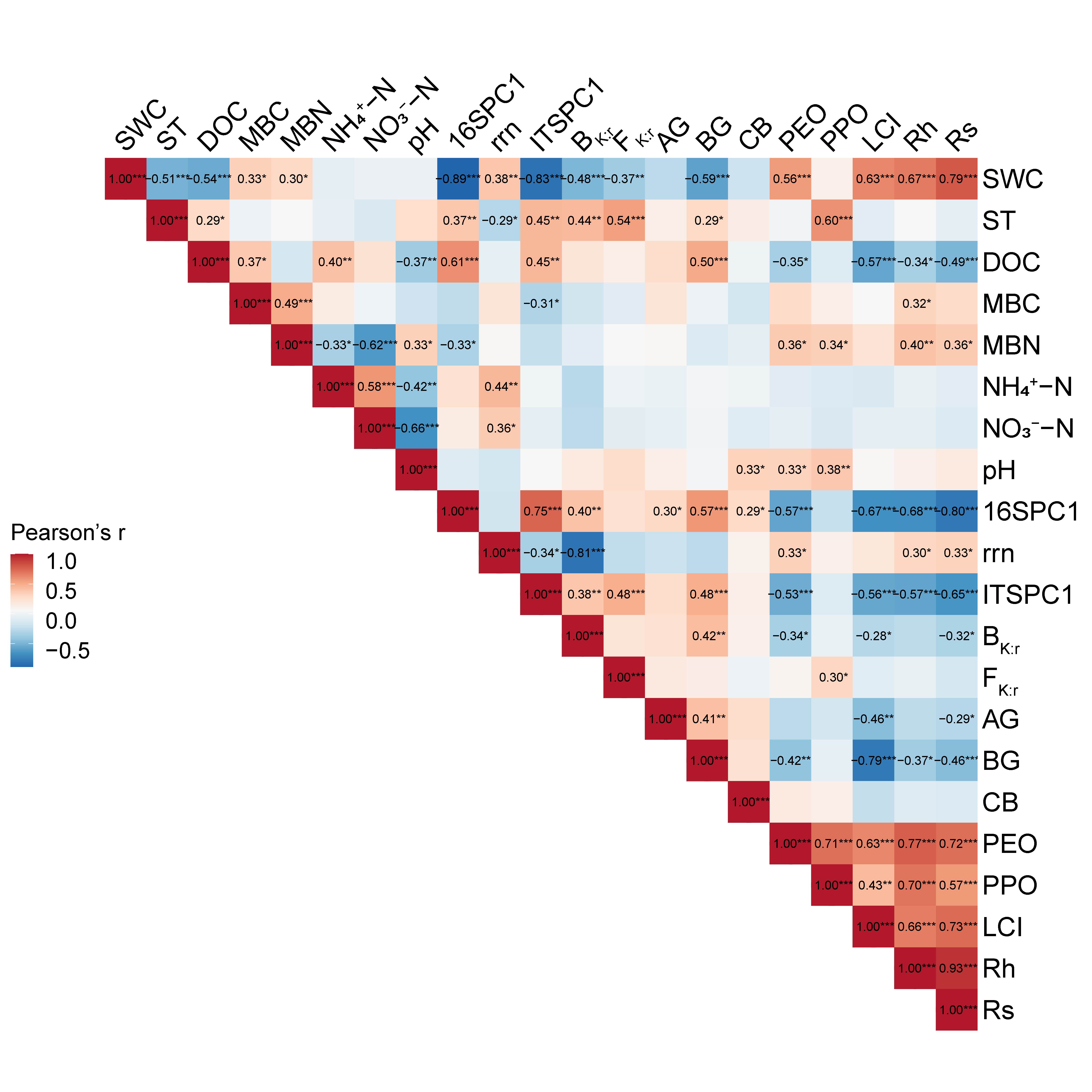
**

**Fig. S3** Pearson’s correlation coefficients between soil properties, microbial properties, enzyme activities, soil heterotrophic respiration, and soil respiration.

SWC, soil water content; ST, soil temperature; DOC, dissolved organic carbon; MBC, microbial biomass carbon; MBN, microbial biomass nitrogen; NH_4_^+^-N, ammonium-nitrogen; NO_3_^-^-N, nitrate-nitrogen; 16SPC1, prokaryotic community composition; ITSPC1, fungal community composition; B_K:r_, the ratio of K- to r-strategists of bacterial phyla; F_K:r_, the ratio of K- to r-strategists of fungal phyla; rrn, abundance-weighted average rRNA operon copy number; AG, α-glucosidase; BG, β-glucosidase; CB, β-D-cellobiosidase; PEO, peroxidase; PPO, polyphenol oxidase; LCI, the lignocellulose index; Rh, seasonal mean heterotrophic respiration; Rs, seasonal mean soil respiration. * 0.01 < *P* ≤ 0.05; ** 0.001 < *P* ≤ 0.01; *** *P* ≤ 0.001
